# Design of a RCT evaluating the (cost-) effectiveness of a lifestyle intervention for male construction workers at risk for cardiovascular disease: The Health under Construction study

**DOI:** 10.1186/1471-2458-8-1

**Published:** 2008-01-03

**Authors:** Iris F Groeneveld, Karin I Proper, Allard J van der Beek, Cor van Duivenbooden, Willem van Mechelen

**Affiliations:** 1 Department of Public and Occupational Health, EMGO Institute, VU University Medical Centre, Van der Boechorststraat 7, 1081 BT Amsterdam, The Netherlands; 2 Body@Work, Research Centre Physical Activity, Work and Health, TNO-VUmc, Van der Boechorststraat 7, 1081 BT Amsterdam, The Netherlands; 3 Stichting Arbouw, La Guardiaweg 4, 1043 DG Amsterdam, The Netherlands

## Abstract

**Background:**

Of all workers in Dutch construction industry, 20% has an elevated risk of cardiovascular disease (CVD). A major risk factor for CVD risk is an unhealthy lifestyle. The aim of our study is to design a lifestyle intervention for construction workers with an elevated CVD risk, and to evaluate its (cost-) effectiveness.

**Methods/Design:**

In a RCT, 692 participants will be randomised to either the control or the intervention group. The control group will receive usual care. For the intervention group, a lifestyle intervention has been designed based on interviews and current literature. The intervention will last 6 months and will comprise 3 face-to-face and 4 telephone contacts, consisting of individual counselling aimed at increasing daily physical activity (PA) and improving dietary behaviour, and/or smoking cessation. Counselling will take place at the Occupational Health Service (OHS), and will be done according to motivational interviewing (MI). Additional written information about healthy lifestyle will also be provided to those in the intervention group. At baseline, after 6 and after 12 months, measurements will take place. Primary outcome variables will be the lifestyle behaviours of concern, i.e. daily PA, dietary intake, and smoking status. Secondary outcome variables will be body mass index (BMI), systolic and diastolic blood pressure, total and HDL blood cholesterol, Hba1c and cardio-respiratory fitness (CRF). Sickness absenteeism and cost-effectiveness will be assessed as well. Multilevel analysis will be performed to compare all outcome measures between the intervention group and the control group.

**Discussion:**

By improving lifestyle, CVD risk may be lowered, yielding benefits for both employee and employer. If proven effective, this lifestyle intervention will be implemented on a larger scale within the Occupational Health Services in construction industry.

**Trial registration:**

Current Controlled Trials ISRCTN60545588

## Background

In the Netherlands, cardiovascular diseases (CVD) are responsible for one third of all deaths each year. Besides to premature death and a decreased quality of life, CVD morbidity leads to high costs for health care and loss of productivity [1]. An unhealthy lifestyle, e.g. smoking, unhealthy diet and/or insufficient daily physical activity PA, is a major cause of CVD [2]. Well-known consequences of an unhealthy lifestyle are overweight, an unfavourable total/HDL cholesterol ratio, and an elevated blood pressure [3,4]. Improved lifestyle may contribute to an improved CVD risk profile. As to smoking cessation, it has been shown that those who have quit smoking quickly reduced the risk of CVD [2,5]. As to diet, there are several ways to decrease the CVD risk. For instance, diminishing intake of saturated fat will lower LDL and total cholesterol [2]. A diet low in salt has a beneficial effect on blood pressure [6]. Fruits and vegetables are rich in micronutrients and fibre and therefore protective against CVD [7]. Moreover, obesity, a major CVD risk factor, may be prevented by lowering calorie intake [8]. As to PA, there is sufficient evidence that regular PA is beneficial for health, not only by preventing weight gain but also by improving cardio respiratory fitness (CRF) [9,10]. In 1995, the Centers for Disease Control and Prevention (CDC) and the American College of Sports Medicine (ACSM) achieved consensus about the level of PA needed for good health. This recommendation stated that every adult should accumulate 30 minutes or more of moderate-intensity PA on most, preferably all, days of the week [11]. Next to this Public Health recommendation on moderate PA, the ACSM stated that each adult should perform vigorous exercise for 20 minutes or more, at least 3 times a week in order to improve CRF [12]. Like in the general Dutch population, an unhealthy lifestyle is commonly seen in Dutch construction industry. Even though the percentage of construction workers that fulfilled the guideline for moderate PA in 2005 (61%) was higher than that of the general Dutch male population (51%) [13], the prevalence of overweight and obesity in the construction industry was considerably higher than in the general Dutch population (64% vs. 51% and 15% vs. 10%, respectively). Only 18% of construction workers fulfilled the guideline for vigorous PA, as compared to 25% of the general Dutch male population. Furthermore, one third of all workers in the construction industry are smokers [14]. Since a substantial part of the ageing population in construction industry has an unhealthy lifestyle, CVD morbidity and mortality in this population is likely to rise in the following years. Sickness absenteeism due to CVD and other chronic diseases may also increase. In collaboration with Arbouw, a national organisation involved in monitoring and improving working conditions and occupational health of workers in construction industry, we will investigate the effectiveness and cost-effectiveness of an individual lifestyle intervention for male construction workers at risk for CVD. The aim of the intervention is to lower the risk for CVD by changing one or more lifestyle behaviours. As the intervention will be focussed on improving three lifestyle topics, i.e. diet, PA, and smoking, we hypothesise that in the intervention group diet will be improved, the amount of PA will be increased, and the number of smokers will be lowered. As these lifestyle components are important CVD risk factors, we further hypothesise that mean BMI, blood pressure, HbA1c and total cholesterol will be lowered and HDL cholesterol will be increased as a result of the intervention. In this article we will describe the design of this study, which has been named 'Health under Construction'.

## Methods/Design

### Study design

In this randomised controlled trial (RCT) we aim to include 692 workers with an elevated CVD risk. The intervention will consist of individually-based lifestyle counselling, plus short written materials about CVD and healthy lifestyle. The control group will receive care as usual. Measurements will take place at baseline, after 6 and after 12 months. The study protocol was approved by the Ethics Committee of the VU University Medical Centre.

### Setting

The study population will be recruited from all Occupational Health Services (OHSs) throughout The Netherlands that perform Periodical Health Screenings (PHSs) in construction industry. A PHS is offered every four years to all construction workers under the age of 40. Workers above the age of 40 are invited for a PHS every two years. All occupational physicians (OPs) and occupational nurses employed at the abovementioned OHSs were invited to apply for the role of lifestyle counsellor in our study. We made a selection based on their motivation, interest in lifestyle, and affinity with the target group.

### Study population

The study population exists of workers in Dutch construction industry who have an elevated risk of CVD. Since less than 10% of workers in construction industry is female, we decided to include only men. Two types of construction workers can be distinguished. More than 75% of all workers take part in the actual construction process ('blue collar workers') and the remaining are mainly involved in planning, supervision en administration ('white collar workers'). Both blue and white collar workers will be included in the study. Their CVD risk will be derived from their PHS results. The PHS consists of a questionnaire to be filled in by the worker at home, and a physical examination, which is performed by an OP or an occupational nurse at the OHS. In the PHS, both work-related health and lifestyle-related health, e.g. smoking, body mass index (BMI), and cholesterol are assessed. Until recently, there was no instrument for determining the workers' risk of CVD based on all risk factors measured in the PHS. For the purpose of this study, we developed such a risk assessment instrument. For this instrument, we used the scores for age, total and HDL cholesterol, blood pressure and smoking, as determined in the Framingham risk score. The Framingham risk score is a widely used sensitive screening instrument for predicting the 10-year risk of coronary heart disease, and is applicable to the US as well as to European populations [15]. Our risk assessment instrument differs from the Framingham risk score because of a lack of data on diabetes. Besides, the risk assessment instrument was elaborated by six additional CVD risk factors, i.e. BMI, alcohol intake, HbA1c, psychological complaints, heart complaints and the amount of weekly PA, measured in the PHS for Dutch construction workers. The cut-offs for these additional CVD risk factors were defined based on expert consensus, recent research and general Dutch standards [16,17]. In Table 1, all inclusion criteria are presented. Of all blue and white-collar workers in Dutch construction industry who will be subject to a PHS between January 2007 and October 2007, those meeting the inclusion criteria will be invited to participate. The invitation letter will be accompanied by a brochure with background information about the study, an informed consent form and a questionnaire. Those who agree to participate, by signing and sending back the informed consent form and the questionnaire, will be included in the study. Exclusion criteria will be 'inability to be physically active', and 'not sufficiently capable of using the Dutch language'.

**Table 1:** Inclusion criteria for the study population.

**Variable**	**Criteria**
Gender	Male
Age	18–65 years
Permission	Signed informed consent
Availability	Available for the study for 12 months following inclusion.
Health status	Having a 10-year risk of coronary heart disease higher than average compared to the same age group according to the Framingham risk score, *and *having *one or more *of the following risk factors:
	• Insufficiently active: Fulfilling none of the Public Health PA guidelines
	• Excessive alcohol use: ≥ 35 glasses of alcohol per week
	• HbA1c ≥ 6.5%
	• Obesity: BMI ≥ 30 kg/m^2^
	• Psychological complaints: Tiredness or stress and/or treated for psychological disorders and/or low motivation to recover from work.
	• Heart-related complaints: Shortness of breath and/or suffering from chest pain and/or diagnosed with or treated for CVD or its predictors, e.g. high blood pressure.

### Randomisation and stratification

After baseline measurements, all participants will be randomly assigned to the intervention or the control group. The study population will be pre-stratified by type of work (blue collar or white collar). Randomisation will take place at the individual level. Using Random Allocation Software (Version 1.0, May 2004, Isfahan University of Medical Sciences, Iran) a random list of letters A and B will be generated for both strata. A research assistant will assign a letter to each of the two groups.

### Sample size calculation

**Figure 1:**
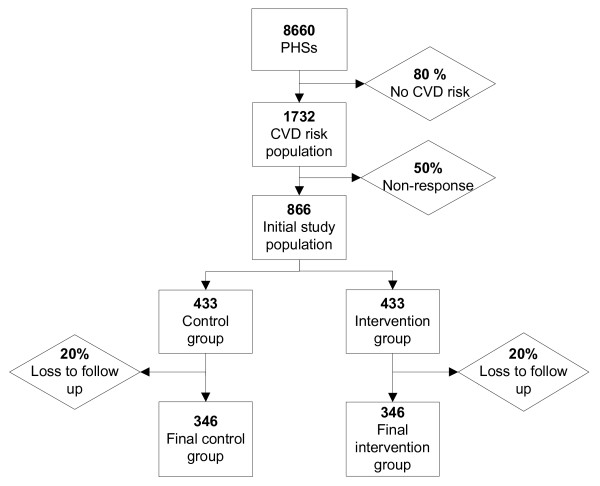
Flow diagram of recruitment of the study population.

The sample size is based on detecting a difference in daily PA according to the guideline for moderate intensity PA. Previous PHSs (2005) showed that 38% of construction workers with an elevated CVD risk was physically inactive according to this guideline. To show a 10% difference between the intervention and the control group after 26 weeks, i.e. 38% inactivity in the control group and 28% inactivity in the intervention group, with a power of 80% and a 95% confidence interval (α = 0.05), 692 persons will be needed (346 in each group). As we expect a loss to follow-up of 20%, 866 persons will be included at baseline. Based on an assumed initial non-response of 50%, we will invite 1,732 workers. In The Netherlands, each month approximately 3,334 workers are screened in a PHS. Based on PHS data obtained in 2006, we expect that on average approximately 667 of them (20%) will fulfil the inclusion criteria. Therefore, we expect that the recruitment period will last at least 3 months. The numbers are presented in Figure 1.

### Blinding

The design of the study (individual counselling versus usual care) makes blinding of participants impossible. Neither the counsellors can be blinded. The research assistant will perform the randomisation procedure, and process the follow-up questionnaires. The principal investigator, who is responsible for the data analysis, will thus be blinded for the allocation. In the invitation letter for follow up, the worker will be asked not to reveal his group status to the occupational nurse, so that the latter will be blinded as well. However, there is no guarantee that the occupational nurses will be blinded in all cases.

### Co-interventions

It is likely that some participants, in the intervention group as well as in the control group, will make use of other CVD risk-related health care, such as medication, other types of treatment, or lifestyle advice by another health care professional. Furthermore, some participants in the control group may start changing their lifestyle during the study, after being confronted with their CVD risk. Such 'co-interventions' cannot be avoided and will therefore be assessed in the questionnaires at baseline and follow-up. Use of additional health care as a result of the counselling, e.g. physiotherapy, dietician advice, or nicotine replacement therapy (NRT), will nor be encouraged neither be discouraged per se since it may have beneficial effects on lifestyle that may not be obtained without the intervention.

### Compliance and loss to follow-up

Except for those who have explicitly stated to leave the study, all participants will receive a questionnaire 26 and 52 weeks after baseline, and an invitation for a health check at the OHS. The number of contacts, as well as loss to follow-up, will be recorded by the research assistant. We will try to avoid or reduce loss to follow-up by various means. For example, each counsellor will have personal contact with his or her own clients. If the client does not show up at the OHS or answer the telephone at the appointed time, the counsellor will call him again to make a new appointment. Furthermore, incentives will be sent to all participants.

### Usual care

Since we aim to assess the effectiveness of a lifestyle intervention as compared to usual care, the control group does not receive the intervention of concern neither any addition to usual care except for the follow-up measurements. Thus, each participant in the control group will receive care as usual, provided by his own OP. Usual care for those with an elevated CVD risk consists of informing the worker about his risk profile. This may be done in a brief oral explanation or by post. In some cases, the OP provides brochures about CVD risk factors and/or lifestyle. Whether further care is provided, such as personal lifestyle advice or referral to the general practitioner (GP), depends on the seriousness of the risk and the OP's usual practice.

### Development of the intervention

#### Interviews

Preceding the study, interviews were held with 14 employees, 6 employers and 9 OPs [18]. The aim of those interviews was to get insight in the workers' current lifestyle, their attitudes towards changing behaviour, their preferences for the type of lifestyle intervention and the feasibility of a lifestyle intervention in construction industry. It appeared that white-collar workers in particular reported a lack of regular PA. Most blue-collar workers said to have daily PA at work. However, hardly any of the interviewed workers reported participating in organised sports. In general, the workers thought to have a healthy diet, but some reported to eat large amounts. Most workers had a positive attitude towards a lifestyle intervention, under the condition that participation in the intervention would be voluntary. Most workers said they would prefer at least one face-to-face contact, preferably at the start of the intervention, with a professional health counsellor. As to the frequency of the contacts, most workers suggested about once a month. They were generally positive about additional counselling by telephone. The majority of the workers interviewed thought that brochures about lifestyle would be helpful, if provided additionally. Furthermore, toolbox meetings at work were suggested as a good opportunity for giving lifestyle advice. Email or SMS were not considered as appropriate media. Some of the workers said that they would appreciate involvement of their wives in the intervention, especially if the intervention would focus on a change in dietary behaviour. The 6 employers who were interviewed emphasised that the intervention should not be pedantic, and that the worker should be confronted with his risk profile and the associated negative health consequences. Moreover, the employers agreed that the person carrying out the intervention should be experienced in counselling and he/she should have experience with the target population. From the interviews with the OPs, it was confirmed that there is neither consensus nor a guideline for lifestyle-related health promotion aimed at workers in the construction industry. All of them were enthusiastic about the initiative of developing a lifestyle intervention. To date, OPs hardly perform lifestyle counselling, because of a lack of time, knowledge, and possibilities for follow-up. They suggested linking the lifestyle intervention to the PHS. According to the OPs, the workers' CVD risk should be communicated in a way that is easy to understand, e.g. visually.

Based on the interviews we concluded that the intervention should preferably be linked to the PHS; contain monthly face-to-face and/or telephone contacts with one and the same counsellor; especially focus on PA in leisure time and the amount of dietary intake; involve the workers' partner if possible. Moreover, CVD risk should be communicated in an understandable way.

#### Literature search

A literature search was performed to gain more insight in the effectiveness of previously implemented lifestyle interventions. In the report of Proper et al (2005), especially individually-based lifestyle interventions appeared to be effective [19]. Since we were interested in smoking, diet and PA behaviour in particular, we looked at studies aimed at these three lifestyle factors. For smoking, it has been shown that a minimal contact behavioural intervention using the stage-of-change concept in Dutch general practice significantly improved self-reported abstinence rates at 12-month follow up [20]. Duration and frequency of counselling in a smoking intervention appeared to have a strong dose-response relation with smoking cessation rates [5,21]. However, even though individual counselling is potentially effective in smoking cessation, success rates for smoking cessation and abstinence may be increased by using NRTs, such as nicotine plasters, gum and spray [5]. Concerning dietary behaviour change, Steptoe et al. (1999) showed that brief dietary behavioural counselling led to decreased dietary fat intake among adults with increased CVD risk in general practice [22]. Furthermore, personalised feedback has been shown to improve attitude and intention, and to significantly increase vegetable intake and decrease fat intake among healthy employees [23]. Finally, evidence was found for the effectiveness of individually-based PA interventions, among sedentary adults in general practice [24], as well as in a workplace setting [25]. Individual counselling can be performed in several ways. In recent years, counselling has become more client-centred. One potential effective and client-centred technique is motivational interviewing (MI). MI is a non-directive, client-centred counselling method, which was originally developed for use in addiction interventions [26]. In recent years, it has also been proven feasible and effective in lifestyle interventions [27-29]. According to Rubak *et al.* (2005), the effect of MI increases with an increasing number of face-to-face encounters [30]. MI is based on five principles, i.e. showing empathy, avoiding discussion, rolling with resistance, supporting self-efficacy, and raising awareness of a dissonance between actual behaviour and behaviour goals. Important non-directive communication strategies used in MI are: Asking open questions, reflective listening and orderly summarizing. Providing information, provocation, and selective confirmation are more directive strategies. In MI a fluent continuum of readiness-to-change is presumed [31]. The transtheoretical model (TTM) of stages of change (SOC) has played an integral role in the development of MI. Another model describing the stages of behaviour change that we found in the literature is the Precaution Adoption Process Model (PAPM) [32]. The PAPM distinguishes seven stages: 1) Unaware of the health issue, 2) Aware of the issue but not personally engaged, 3) Engaged in the issue and deciding what to do, 4) Having decided not to act, 5) Planning to act but not yet having acted, 6) Initiating the behaviour, and 7) Having maintained the behaviour over time. In contrast to the TTM, the PAPM distinguishes between stages 1 and 2 and between stages 4 and 5. In a stage-based intervention, in general, persons unaware or unengaged may need to be made more aware of the risks of their own health situation and the possibility of behaviour change. They can be informed, advised and encouraged. With persons in the decision stage, barriers and benefits of behaviour change should be discussed, and 'the positive' should be accentuated. Persons in the preparation stage need to set short-term goals that are acceptable, accessible and effective, taking into account past experiences. Persons who just initiated behaviour change deserve affirmation for what they have accomplished and encouraging comments. They may need assistance in possible revision of their plans. In this stage, anticipation on possible future barriers may also be necessary. The same strategy holds true for persons who have already maintained behaviour over time [33]. Persons who have relapsed into former behaviour need to be guided through the stages again. Persons who have decided not to change a certain (sub-) behaviour may decide to change another (sub-) behaviour. To achieve behaviour change, relevant determinants of behaviour will have to be addressed in the intervention. From the literature we learned that attitude, self efficacy, social influence (the principal components of the ASE-model [34,35]), intention [34,36], environment, and habitual behaviour [37] are important behavioural determinants. Finally, we looked at literature about risk communication. Several studies have shown that the explanation of CVD risk to the patient or client should be clear and easy to understand, to prevent confusion or fear [38] and to enable the client to make a well informed choice about if and what he wants to change [39]. Based on the literature, we decided to develop an individually-based lifestyle intervention, comprising frequent contacts, applying MI, with the PAPM as a basis. Next to the SOC, behavioural as well as environmental determinants will be taken into account during the counselling sessions. The workers' CVD risk will be explained to him in an understandable way.

### Outline of the intervention

The intervention will last 6 months, since this is the minimum amount of time to achieve maintenance of changed behaviour. For the reasons described above, MI will be used as a counselling method. Based on the evidence for the effectiveness of frequent contacts, and on the preferences of the interviewed workers, 3 face-to-face and 4 telephone contacts will be scheduled. The first contact will always be face-to-face. The duration of a face-to-face contact will be 45–60 minutes. The telephone calls will take 15–30 minutes. Face-to-face contacts will preferably take place after participant's working hours (that is: after 3 pm) at the OHS. If a participant explicitly states that he does not want to visit the OHS, the counsellor may arrange a home visit. As suggested by the interviewed workers, the wife or partner of the participant will be invited to accompany the participant to the face-to-face meeting. Additionally, written materials will be provided. The intervention will be performed by 24 counsellors (occupational nurses and OPs) who have been trained for the purpose of this study. Each participant will be in contact with one specific counsellor during the entire intervention. Each participant in the intervention group will be allocated to a counsellor in his own region of residence. The counsellor will strive to arrange the first contact within the first week after inclusion, and arrange further contacts each month, with a margin of one week. The last telephone contact should take place in the 25^th ^week after inclusion at the latest. The schedule for each participant in the intervention group is presented in Table 2.

**Table 2:** Contacts schedule for each participant in the intervention group.

**T in weeks**	**Event**
0	Invitation for first face-to-face encounter.
1	Face-to-face contact
4	Telephone contact
8	Face-to-face contact
12	Telephone contact
16	Telephone contact
20	Face-to-face contact
23	Telephone contact
26	First follow-up measurement
52	Second follow-up measurement.

### Training of counsellors and pilot study

All counsellors were trained in MI by an MI- expert. Before the training took place, they were asked to read a short introduction to MI and background information about healthy lifestyle [40], which was sent to them by the principal investigator. In a 3-day course they learned the principles of MI and practiced by means of role-plays, after which they gave feedback to each other. In a pilot study among a small group of construction workers (n = 19), half of the counsellors had a meeting with one or two workers. These counsellors recorded their conversation(s) in the pilot study on audiotape. The tapes were evaluated on the 3^rd ^training session, which was held 6 weeks after the first two-day training session. In this 3^rd ^session, difficulties experienced during the pilot were discussed. Further aims of the pilot study were to assess both the counsellors' and the workers' opinions about the face-to-face contacts, and to test the questionnaire and 8 different brochures about CVD and lifestyle. After the pilot study, comments and suggestions for improvement of the intervention were assessed by the principal investigator by means of evaluation forms. Besides, 7 randomly chosen participants were personally interviewed about their experiences in the pilot. All workers and counsellors were positive about the face-to-face contacts. After the pilot, no major changes needed to be made in the study protocol. As suggested by the participants of the pilot study, the number of brochures to be provided was reduced.

### Counselling protocol

Using MI, the counsellor will guide the participant through the process of becoming aware of the health problem, changing behaviour and maintaining the changed behaviour, as described in the literature. The following steps will be undertaken to reach these goals.

#### Step 1: Introduction of health problem

In the first face-to-face encounter, the counsellor will explain the goals and procedure of the intervention. He or she will discuss the participants' knowledge about CVD risk factors and health consequences, as well as his awareness of his own risk. A personal risk profile, on which the participant's values on five important risk factors (BMI, systolic blood pressure, total cholesterol, days per week involved in PA of moderate intensity, smoking) are visually represented, will be given to the participant and will be explained in a clear way. The counsellor will not move on to the next subject until the worker understands this information. Current lifestyle and family history will be discussed, to get a complete insight in the participants' lifestyle and health status.

#### Step 2: Choosing type of intervention

Two types of intervention will be offered: 1) an energy balance intervention and 2) a smoking cessation intervention. Together with the counsellor, the smoker will choose one of the two intervention types, based on his personal risk profile and his own preferences. He will be encouraged to make his choice during the first meeting, and to stick to his first choice. However, if later on during the intervention, the participant desires to switch intervention types or if he prefers to address both smoking and energy balance, this will be possible. In case a participant is not ready make his choice in the first meeting yet, e.g. because he is confused by his risk profile, he will be allowed to make his choice during the 2^nd ^contact. In the energy balance intervention, both diet and PA will be addressed. Depending on the current PA and dietary behaviour of the participant, the focus will be on diet or daily PA. In improving dietary and PA behaviour, sub-behaviours will be chosen to address, which will be relevant to the participant and feasible, e.g. reducing fat intake or cycling in leisure time.

#### Step 3: Exploring ambivalence

By discussing advantages and disadvantages of a current sub-behaviour (e.g. eating too many snacks, not participating in any sports) as well as of the 'desired' healthy behaviour, ambivalence may be raised. On a 'decisional balance quadrant', the worker will fill in the 'pros of his current behaviour', 'cons of his current behaviour', 'pros of behaviour change' and 'cons of behaviour change'. In doing so, he may become aware of the discrepancy between his current behaviour and the desired behaviour, possibly leading to a change in attitude towards the desired behaviour. As a result, he may realise that it is worth the effort to change his current behaviour.

#### Step 4: Determining readiness, willingness and ability

In step 4, the participant will be asked to indicate his perceived *importance *of change, his perceived *confidence *in his ability to change, and his *readiness *to change on a scale from 0 (not at all) to 10 (highly) [41]. The counsellor will ask why he did not choose a lower number, and what should be done to get him higher up the scale. In doing so, the counsellor and participant may find out which of these items is the most important barrier for change, so that special attention can be paid to that particular item.

#### Step 5: Goal setting

Social influence, the workers' environment and other factors that may influence behaviour will be discussed to facilitate goal setting. At the end of each meeting, a specific short-term goal will be defined by each participant and his counsellor, e.g. visiting the sports centre, reducing the number of snacks per week, or informing family and friends about the intended lifestyle change. This goal will need to be accomplished before the next meeting. In addition, after the first meeting one or more long-term goal(s) will be defined, for example loosing 20 pounds or quitting smoking. These goals need to be accomplished within six months.

Steps 1, 2, 3 and 4 will be discussed preferably in the first session. In the subsequent contacts, progress and obstacles for change will be discussed. Depending on the participants' situation, attitude, social influence, self-efficacy, environmental factors, habits or other determinants may be discussed, to facilitate the process of behaviour change. A new short-term goal will be set after each contact. If the participant asks for information about healthier lifestyle or suggestions for implementation, e.g. where to find a good fitness centre or how to prepare a low-calorie meal, the counsellor will provide this information. A small number of participants may not be of Dutch origin. Their lifestyle and especially dietary habits may be different from the Dutch white Europeans [42]. This will be taken into account by the counsellor when giving advice. The counsellor will stress the importance of incorporating the healthy behaviour into daily life, since this is the best way to achieve maintenance. In case a participant relapses into previous behaviour, the counsellor will guide him through the stages again, taking into account the problems of the past experience [5,31,33].

### Registration forms

During the first contact the counsellor and participant will complete a sheet that contains a summary of what has been discussed in steps 1 and 2, the decisional balance quadrant, the 10-point scales about importance, confidence and readiness, the first short-term goals and the long-term goal(s) (step 3, 4 and 5, respectively). The participant will take this sheet home as a reminder of what was discussed. Further, after each contact, the counsellor will fill in the location and duration of the contact, a summary of the topics discussed, and the (new) short-term goal on a short form. All sheets and forms will be sent to the research assistant, who is then able to check if and how the contacts are taking place.

### Written information

The counsellor will provide several brochures to each participant. The brochure 'Cardiovascular diseases' contains general information on lifestyle-related ailments of the heart, the brain and the vessels. The participants in the 'smoking cessation' intervention will receive a brochure from Stivoro, a Dutch organisation for education about the risks of smoking, describing benefits of smoking cessation, how to prepare a quit attempt, how to prevent relapse, et cetera. Participants in the 'energy balance' intervention will receive a brochure about PA, including guidelines and a training schedule. Furthermore, they will receive four brochures about healthy diet from the Dutch Heart Foundation and the Dutch Nutrition Centre: 1) A brochure with the dietary guidelines of the Dutch Nutrition Centre promoting a varied diet, low in saturated fat and rich in fruits, vegetables and bread, 2) A leaflet describing products high in saturated fat and their low-fat alternatives, 3) A leaflet with the caloric values of the most common food products, 4) A brochure with recipes for healthy meals.

### Incentives

Incentives will be handed out to all participants in the intervention group as well as those in the control group, to make participation more attractive and to avoid loss to follow-up. After 3 months, a t-shirt with the study logo will be sent. Along with the invitations for both follow-up measurements, a lottery ticket will be sent. Among the participants who attend the 2^nd ^follow-up measurement (T = 52 weeks), a heart rate measurement instrument will be raffled. At the same time, a 3-day stay in a bungalow park can be won by the participant who best completes a slogan. At the end of the second follow-up measurement, a step counter will be handed out to all participants.

### Quality assessment

To assess the quality of MI during the intervention, the Motivational Interviewing Treatment Integrity code (MITI) protocol will be applied to two face-to-face contacts of half of the counsellors during the first three months of the intervention. For this purpose, each counsellor will be asked to tape record 2 face-to-face contacts. The principal investigator will randomly choose a 10-minute lasting fragment from each tape, and make a transcript of the verbal communication on paper. Two independent MI-experts will code these fragments by using the MITI protocol [43]. The coding provides two scores: a 'global score' and a 'behaviour count'. The global scores refer to the entire verbal interaction between the participant and the counsellor. A global score for MI spirit as well as for MI empathy will be assigned as a single number on a 7-point scale. The behaviour count is a one-figure rating applied to the separate MI techniques, e.g. reflective listening and asking open questions. Any utterance may be assigned one of 6 primary behaviour codes. After coding, the counsellor will get feedback on his/her performance of MI.

### Measurements

Baseline data will be obtained from the PHS and the additional questionnaire. Six months after inclusion, a follow-up measurement will take place. Twelve months after inclusion, the second follow-up measurement will take place.

### Primary outcome measures

#### Physical activity

The main outcome measure for PA is whether the participant has started to fulfil one or both of the Public Health recommendations for PA. If so, he is considered to have changed from physically inactive to physically active.

On the PHS questionnaire, he will fill in how many days per week he usually is moderately physically active for more than 30 minutes. From this item, fulfilling the Public Health recommendation for moderate PA can be determined. Furthermore, he will be asked how many days during the last month he has performed vigorous physical activities that lasted long enough to make him sweat. By this item, fulfilling the Public Health recommendation for vigorous PA can be computed. Another outcome measure for PA is the frequency, duration and intensity of PA in leisure time. This will be assessed using the Short QUestionnaire to ASsess Health enhancing physical activity (SQUASH), which was shown to be a fairly reliable and valid questionnaire (*r*_*Spearman *_0.58 and *r*_*Spearman *_0.45 respectively) [44]. The SQUASH was designed to measure four clusters of PA, i.e. commuting activities, household activities, activities at work and school, and leisure time activities. For the purpose of this study, only commuting activities and leisure time activities, i.e. walking, cycling, gardening, odd jobs and sports will be measured, as these activities are considered relevant for the target group. The participant will be asked to fill in how many days per week and how many minutes per day he usually spends on these physical activities, during a usual week within the past month. The level of exertion for each type of activity will have to be indicated as well. The level of exertion for sports, gardening and odd jobs is classified as light, intermediate, and vigorous. The level of exertion for walking and cycling is classified as slow, intermediate, and fast. From the SQUASH, metabolic equivalents (METs) can be estimated. Light activities are rated as 2.0 to < 4.0 METs, moderate activities are rated as ≥ 4.0 to <6.5 METs, vigorous activities are rated as ≥ 6.5 METs [44]. The cluster items about household activities will be left out of the SQUASH questionnaire, as we expect these activities not to be major contributors to daily PA in this population of male construction workers. In addition, the SQUASH items about activities at work and school were not considered suitable for our population. The level of exertion, i.e. light, intermediate, or vigorous, of the daily activities at work will be assessed using a single question derived from the TNO questionnaire PA at work [45], which has not been tested for validity and reliability yet.

#### Dietary intake

Daily dietary intake will be assessed for the following food groups: wholegrain and white bread (slices), dinner portions (spoons), regular soft drinks and alcohol (glasses), *light *soft drinks (glasses), sweet and salty snacks (pieces), vegetables and fruits (pieces or portions), fish and crustaceans (portions). Of these food groups, average intake on days per week and slices/spoons/glasses/pieces/portions per day during a usual week in the past month will be indicated. High intake of regular soft drinks, alcohol and snacks is considered as unhealthy dietary behaviour, whereas high intake of light soft drinks, vegetables, fruits and fish is considered as healthy dietary behaviour. The questions about fruits and vegetables were derived from the short questionnaire for measuring fruit and vegetable intake, developed by Maastricht University [46]. This questionnaire was shown to be sufficiently reproducible (*r*_*Spearman *_0.79 after 1 year) and appeared to be suitable for ranking individuals according to their consumption of fruits and vegetables and according to changes in their consumption, at least in females. As we learned from the interviews, total daily food intake in this population is relatively high. Therefore, the items 'bread' and self-rated 'portions for dinner' were added to assess the daily 'amount' of food intake. All of these food groups may be targeted in the energy balance intervention, depending on the participants' actual diet.

#### Smoking

Current smoking status (smoker or non-smoker) will be assessed by a single item.

#### Biomedical variables

BMI (kg/m^2^), systolic and diastolic blood pressure (mmHg), HDL-cholesterol, total cholesterol (mmol/l) and HbA1c (%) will be assessed by an occupational nurse at the PHS. First, weight will be measured on a digital balance, without shoes and jacket. After that, blood pressure will be measured once, by manual inflation, while the worker is seated. In most OHSs, a Maxi Stabil 3 measuring instrument (Speidel & Keller) will be used for this purpose. Finally, for the determination of HDL-cholesterol, total cholesterol and HbA1c levels venous blood will be drawn from the lower arm and transported to the laboratory for analysis. For each of these 3 measures 1.6 ml blood will be used. As described in the introduction, elevated BMI, blood pressure and cholesterol are indicators of CVD risk. HbA1c, a measure of haemoglobin glycosylation, is an indicator of the mean blood glucose level of the past 6–8 weeks [47]. Elevated HbA1c was shown to be an independent risk factor for coronary heart disease in persons with and without diabetes [48].

### Secondary outcome measures

#### Cardio respiratory fitness

CRF will be measured in an indirect way, by means of a predictor rule that was developed by an international group of experts in the fields of epidemiology, fitness assessment and preventive medicine, and has been shown to be a reasonably accurate measure of VO_2_max [49]. Using this prediction rule, VO_2_max is estimated from BMI, age, gender, resting heart rate (HR) and self reported data on habitual PA levels. The outcome of this prediction rule is indicative of a person's maximal workload expressed as METs; a measure for CRF. Resting HR is not assessed in the PHS and can neither be added for practical means. Therefore, it will be measured by the counsellor at the first face-to-face encounter. At the follow-up health check-ups after 6 and 12 months HR will be measured again, only in the intervention group. For measuring resting HR a heart rate instrument (Polar S610, Polar Electro Oy, Finland) will be used. The participant will take off his shirt and lie down on examination bench. The Polar belt will be tied around his breast. The counsellor will read the HR from the Polar watch, 10 seconds after it has reached the lowest frequency, and record this figure on the registration form.

#### Sedentary activities

In leisure time, the study population is relatively sedentary. Sedentary behaviour is characterised by pursuits that require minimal amounts of energy, e.g. television watching and computer use. Several studies have shown that sedentary behaviour is, independently from the PA level, a risk factor for CVD [50,51]. Using the AQUA questionnaire, previously used by Slootmaker *et al.*[52], we will assess the days per week and minutes per day spent on television watching, computer use, sitting in a car or other sedentary activities in a usual week during the past month.

#### Stage of change

The 'stage of change' items in the questionnaire are based on the stages as defined in the PAPM. The stage of change for *eating less or healthier *as well as the stage of change for *becoming more physically active *will be measured using a single question with 5 outcome categories, of which the participant needs to choose the most appropriate. For PA, these categories are: "I have never thought about becoming more physically active"; "I have thought about becoming more physically active, but I do not know (yet) whether I will do so"; "I have decided not to become more physically active"; "I have decided to become more physically active but I am not currently doing so (yet)"; "I think I am already physically active enough". The questions were derived from Wammes *et al.*[53], who applied the PAPM to a non-obese population in a study aimed at weight gain prevention, following Weinstein's staging algorithm. The validity and reliability of this questionnaire have not yet been tested.

#### Behavioural determinants

As described before, attitude, social influence and self-efficacy are important determinants of behaviour (change) and will be addressed in the intervention. Therefore, attitude (on a 5-point Likert scale ranging from *very bad *to *very good*), self-efficacy (on a 5-point Likert scale ranging from *very difficult *to *very easy*) and social influence of partner, relatives, colleagues or friends (on a 5-point Likert scale ranging from *not at all *to *very much*) will be measured. The same determinants will be measured for *becoming more physically active*. The items about attitude and self-efficacy were derived from Oenema *et al.*[54].

#### Sickness absenteeism

Each participant will be asked to fill in 'total days absent from work' in the past 2 months. Since the difference between registered and reported sick leave was shown to increase with an increasing recall period, Severens et al. (2000) suggested a recall period of no more than 2 months [55]. Therefore, besides the questionnaires at baseline, 6 and 12 months, an additional short questionnaire measuring sickness absenteeism only will be sent to all participants after 2, 4, 8 and 10 months. Participants will not only be asked to report their sickness absenteeism in general, but also their sickness absenteeism due to sports injury, as sports injuries may result form the intervention.

#### Cost-effectiveness

Next to investigating the effects of the intervention, the costs of the intervention will be compared to the effects. All costs for the development of the intervention, i.e. training and printing of brochures, as well as for the implementation of the intervention, i.e. (previously defined) reimbursements for participant's travelling expenses, counsellor's face-to-face and telephone contacts and occupational nurses' health checkups, will be recorded by the principal investigator. Incremental costs of the intervention group compared to the control group will be divided by incremental effects for each of the biomedical effect measures, e.g. BMI, total cholesterol and systolic blood pressure, separately.

#### Process evaluation

Next to an effect evaluation, the process of the intervention will be evaluated in three ways. First, at post-test the participants in the intervention group will be asked for their opinions about 1) the intervention as a whole; 2) the counsellors' competence; 3) the visits to the OHS, the telephone calls and the written materials; and 4) the effect of the intervention on their own behaviour change. Second, by asking a random sample of the counsellors to tape-record two of their conversations, the quality of MI will be assessed, as described before. Third, by means of forms filled in by the counsellor after each conversation, the amount and duration of each conversation will be assessed, the main subject of each conversation, whether long-term goals have been achieved, et cetera.

### Data analysis

On all outcome measures, intention-to-treat analyses will be performed. Both crude and adjusted linear and logistic regression analyses will be performed. In doing so, analysis for covariance will be conducted adjusting for the outcome variable measured at baseline. In the adjusted model, other potential confounders will be included as covariate, such as age and work activities. Furthermore, effect modification, e.g. by family history, will be checked. First of all, data will be analysed for the whole intervention group, that is, irrespective of the intervention received. Secondly, subgroup analysis will be done among those having received the smoking or the energy balance intervention. As all participants will have to indicate at baseline which lifestyle behaviour they prefer to change, the participants in each intervention subgroup will be compared to those in the control group having indicated the same type of intervention preference. Cost-effectiveness ratios will be calculated by dividing the difference between the mean total costs between the two study groups by the difference in the mean effects. For each outcome measure used in this evaluation, cost-effectiveness ratios will be presented graphically on a cost-effectiveness plane. To calculate the confidence intervals for the ratios, bootstrapping will be done. For all analyses, a significance of 0.05 will be applied. On the process evaluation, qualitative analyses will be performed.

## Discussion

This lifestyle intervention was developed as a tool for prevention of CVD among those with an elevated risk. The content of the intervention is evidence-based and tailored to the target group. Furthermore, the intervention will exclusively be aimed at persons at risk for CVD, who may more likely be aware of the need to change behaviour. Counselling will be performed by professionals experienced in face-to-face counselling of this target group. Based on the literature and interviews with relevant stakeholders we designed a structured counselling protocol, incorporating MI strategies. Counsellors will raise awareness of the health problem and discuss determinants of behaviour change, to facilitate the participant in increasing importance, ability and readiness to change, and in changing actual behaviour. By applying the MITI, we will be able to report on the quality of MI in the intervention. The written information that will be given to the participants contains practical information and is easy to understand.

Several limitations of this study can however be mentioned. First, stress and other psychosocial problems will not primarily be addressed in the intervention, even though these factors are positively related to CVD risk. The counsellors were not trained in solving psychosocial problems. Stressful circumstances such as job strain, high work demands, and low job control that appear to be a barrier for behaviour change will however be discussed. Secondly, the intervention does not take place at company level. Therefore, the workplace environment cannot be changed, even though the environment has been shown an important determinant of health behaviour [37]. Furthermore, in an individually-based intervention study participants may lack the support of other participants or their employer, and the suggested 'toolbox meeting' cannot be used as a medium for the intervention. Finally, in the PHS only a limited number of variables is measured. Therefore, CRF will be derived from an indirect instrument that may be less accurate in predicting VO_2_max than a step-test or treadmill-test. For the same reason, waist circumference will not be assessed, even though abdominal obesity has been proven to be an important indicator for CVD risk [56,57].

Studying the effects of this intervention is important, as it aims at a serious and increasing health problem. This type of intervention has not been evaluated in this specific setting and in this target group yet. If proven effective, the employee will benefit from an improved lifestyle and a reduced CVD risk, and the employer may benefit from healthier workers and lower absenteeism. If proven cost-effective, in the future the OHSs in construction industry may implement this intervention on a larger scale.

## Competing interests

The author(s) declare that they have no competing interests.

## Authors' contributions

IG designed the intervention protocol and wrote the manuscript. KP wrote the initial study protocol and was involved in preparations for the study. KP, AB, CD and WM provided intellectual input and had a role in supervision. All authors have read and approved the final version of the manuscript.

## Pre-publication history

The pre-publication history for this paper can be accessed here:


